# An Addendum to the Chemiosmotic Theory of Mitochondrial Activity: The Role of RNA as a Proton Sink

**DOI:** 10.3390/biom15010087

**Published:** 2025-01-08

**Authors:** Ramin M. Farahani

**Affiliations:** 1 School of Medical Sciences, Faculty of Medicine and Health, The University of Sydney, Sydney, NSW 2006, Australia; ramin.mostofizadehfarahani@sydney.edu.au; 2 IDR/Research and Education Network, WSLHD, Westmead, NSW 2145, Australia

**Keywords:** chemiosmosis, proton motive force, proton sink, RNA

## Abstract

Mitochondrial ATP synthesis is driven by harnessing the electrochemical gradient of protons (proton motive force) across the mitochondrial inner membrane via the process of chemiosmosis. While there is consensus that the proton gradient is generated by components of the electron transport chain, the mechanism by which protons are supplied to ATP synthase remains controversial. As opposed to a global coupling model whereby protons diffuse into the intermembrane space, a localised coupling model predicts that protons remain closely associated with the lipid membrane prior to interaction with ATP synthase. Herein, a revised version of the chemiosmotic theory is proposed by introducing an RNA-based proton sink which aligns the release of sequestered protons to availability of ADP and Pi thereby maximising the efficiency of oxidative phosphorylation.

## 1. Introduction

**Figure 1:**
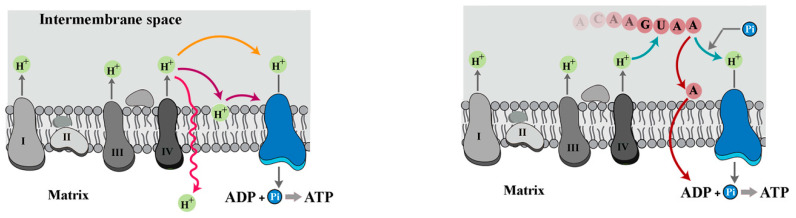
Overview of the revised chemiosmotic theory. The left schematic image shows three competing fates of generated proton via ETC in the original version of chemiosmotic theory (diffusion into solvent, diffusion via lipid membrane, leakage through lipid membrane). The right schematic image demonstrates a putative RNA-based proton sink which releases the trapped protons and ADPs (mixed with other NDPs) upon receiving feedback from inorganic phosphate.

ATP synthesis via mitochondrial F_0_F_1_-ATP synthase requires activity of the electron transport chain to maintain an electrochemical proton gradient across the mitochondrial inner membrane [1]. In the global coupling model of chemiosmosis, originally proposed by Mitchell, protons are initially transported into the intermembrane space via components of the electron transport chain, and this generates a proton motive force to be utilised by F_0_F_1_-ATP synthase for synthesis of ATP [2]. Announcement of the generalised coupling version of chemiosmosis sparked research aimed at providing an explanation for how the expelled protons could be retrieved from the “entropic ocean” of the aqueous phase of the intermembrane space (IMS). Gradually, evidence accumulated to support a localised coupling version of chemiosmosis whereby transfer of protons to F_0_F_1_-ATP synthase occurs via the lipid bilayer (i.e., mitochondrial inner membrane) [3], rather than the bulk solvent phase of IMS. Subsequently, rigorous measurements revealed that while protons diffuse through both solvent as well as the lipid bilayer, the speed of diffusion through the membrane is an order of magnitude higher than the water phase [4,5] (Figure 1). Another hurdle in diffusion of protein from the “source” to the “sink” is permeability of the mitochondrial inner membrane to protons, which increases non-linearly with respect to proton motive force (Δp) [6,7]. According to chemiosmosis theory, a proton motive force of 210 mV is required to activate synthetic activity of F_1_F_0_ ATP synthase [8,9]:

∆p=∆E−60∆pH≈210 mV

This implies that a pH differential of 3.5 units or a membrane potential of 210 mV or a combination could trigger ATP synthesis [9]. However, proton leakage through the inner membrane increases steeply (i.e., non-linearly) above a threshold value of Δp triggering a futile proton cycle and depressing Δp. This non-ohmic behaviour is further compounded by the intrinsic permeability of phospholipid membranes to protons [10]. From a theoretical perspective, a “transient proton sink” that sequesters protons in the vicinity of the inner membrane prior to transfer to the main sink, F_1_F_0_ ATP synthase, could reduce the impact of futile proton cycling by supplying a large quantity of trapped protons in a restricted temporal window. A transient proton sink is not required just to minimise the impact of a futile proton cycle. The critical role of a “transient sink” is also foreshadowed by the fact that solvation of protons to H_3_O^+^ renders it difficult if not impossible to reclaim the sequestered protons due to a high de-solvation penalty of >500 meV [11]. Following this line of reasoning, it would be surprising if a requirement for a transient proton sink in ATP synthesis remains merely a theoretical curiosity. *Escherichia coli* and *Bacillus brevis* appear to significantly benefit from employing a transient proton sink that emerges upon adhesion onto a negatively charged surface. The negatively charged surface operates as a transient proton sink amplifying the proton motive force and increasing ATP level two to five times compared to ATP levels in corresponding planktonic cells [12]. Underlying this phenomenon of enhanced ATP production is enrichment of H^+^ in the vicinity of a negatively charged adhesion surface, which leads to amplification of Δ*p* [12].

Aside from the dynamics of proton diffusion in chemiosmosis as outlined by Morelli et al. [13], the requirement for a transient proton sink becomes more obvious upon focusing on other substrates of ATP synthesis, ADP, and inorganic phosphate (Pi). A key assumption of the chemiosmosis theory is that the establishment of Δ*p* coincides with the availability of ADP and Pi. Yet, no apparent mechanisms are observed within the theoretical framework of chemiosmosis to explain how the generation of Δ*p* by the electron transport chain (ETC) is synchronised to the provision of substrates for ATP synthesis. In the absence of such synchronicity, not only the efficiency of ATP synthesis will be reduced but also a sustained shift to acidic pH within IMS (due to unconsumed protons) could potentially polarise zwitterionic membrane phospholipids [14] and compromise the integrity of mitochondrial membranes. In an ideal scenario, a putative transient proton sink in IMS would sequester the protons temporarily, an activity that would be reversed upon receiving direct feedback from ADP and Pi, thereby triggering a release of the sequestered protons. Therefore, proton leakage and membrane damage will be minimised by aligning the release of protons to availability of ADP and Pi (Figure 1). Based on emerging evidence [15], it appears that an RNA-based proton sink satisfies the requirements of such an ideal proton sink.

## 2. RNA: The Dark Matter of Mitochondrial Energetics?

It was recently shown that RNase-A-mediated degradation of RNA within IMS leads to ATP synthesis despite stalled activities of the citric acid cycle and the electron transport chain [15]. Underlying this phenomenon is an abrupt reduction in pH due to the release of protons trapped by RNA within IMS and an associated emergent Δp that triggers a spike in ATP synthesis, even when the ATP synthase is in a hydrolytic mode [15]. As expected, mitochondria exhibit a lower capacity for ATP synthesis after the degradation of RNA in IMS [15]. A complementary (but unrelated) observation corroborating the proton-trapping capacity of RNA within IMS occurs during neural differentiation. Upon induction of differentiation, mitochondria transiently fuse with the nuclear membrane and import polyadenylated nuclear-encoded RNAs into IMS, an event that expands the RNA-based proton sink, leading to a collapse of Δp and inhibition of ATP synthesis [15]. Collectively, the evidence suggests that negatively charged RNA within IMS operates as a transient proton sink, amplifying the efficiency of ATP synthesis. This function of RNA is similar to the role played by a negatively charged adhesion surface in enhancing the efficiency of ATP synthesis in *E. coli* and *B. brevis* [12], albeit with a further layer of sophistication. Mitochondria synthesise RNA within IMS in a DNA-independent manner by integrating input from two key substrates of ATP synthesis, ADP and Pi.

**Figure 2:**
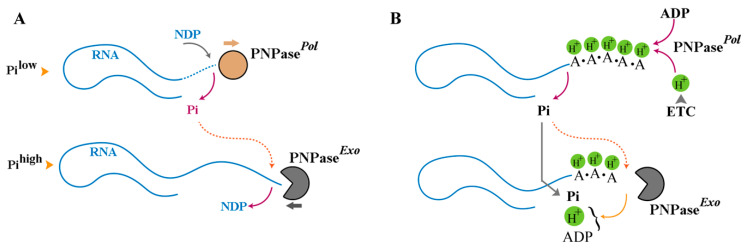
Phosphate-dependent bimodal activity of PNPase. (**A**) At a low phosphate concentration, PNPase functions as a polymerase, converting NDPs to NMPs and lengthening the RNA while releasing the Pi. Contrarily, at a high phosphate concentration, PNPase functions as an exonuclease degrading RNA and utilising the Pi to convert NMPs to NDPs. (**B**) In a synthetic mode, PNPase expands the poly(A) sink and associated protons. Upon switching on the exonuclease activity, ADP is regenerated via phosphorolysis and trapped proton are released to provide the key substrates for ATP synthesis together with the residual Pi.

The key enzyme responsible for synthesis of RNA within IMS is polynucleotide phosphorylase (PNPase) [16]. This enzyme exhibits a dual functionality regulated in a phosphate-dependent manner. PNPase drives 5′→3′ polymerization of RNA in a low phosphate condition, whereas the enzyme catalyses 3′→5′ phosphorolysis of RNA under a high phosphate concentration [17,18] (Figure 2). In a synthetic mode, PNPase operates in a DNA-independent mode [18] and exhibits a preference for ADP as a substrate [19]. As such, in ADP-rich mitochondrial IMS (estimated concentration of ADP ≈ 25 μM [20]), the enzyme synthesises poly-(A) homopolymers in a DNA-independent manner within IMS [15].

The mitochondria-intrinsic synthesis of poly(A) homopolymers by PNPase not only expands the RNA-based proton sink in a nucleus-independent manner but also enriches adenines as a structural component of the sink. This gives rise to a latent Δ*p* (proton motive force) and a reserve adenine pool, two key components required for ATP synthesis by F_0_F_1_-ATP synthase. As the concentration of inorganic phosphate (Pi) within IMS increases beyond a threshold level (Figure 2A), the synthetic activity of PNPase ceases and the enzyme catalyses 3′→5′ phosphorolysis of poly(A) RNA [17,18] liberating the reserve of ADP and the trapped H^+^. While some variability is observed between various species, a threshold Pi concentration of >0.1 mM triggers exonuclease activity of human PNPase [21]. In the proposed revised model, the reserve ADP and the latent H^+^ are liberated by PNPase only as [Pi] reaches a threshold level thereby assuring that all three components required for ATP synthesis (ADP, Pi, H^+^) are supplied to F_0_F_1_-ATP synthase in a synchronised manner (Figure 2B). In support of the proposed role of PNPase in regulating mitochondrial ATP synthesis, Chen et al. demonstrated that reduced PNPase activity impairs mitochondrial electrochemical membrane potential, decreases respiratory chain activity, and suppresses ATP production [22]. In this study, PNPase knockdown resulted in a three- to fourfold loss in Δp and a >50% reduction in ATP production compared to control cells maintained under normal growth conditions [22]. Interestingly, deployment of adenine as a structural component of the proton sink in IMS offers the compounded benefit of enriching a key energetic substrate together with an effective proton acceptor as discussed in the following section.

## 3. RNA as a Polymeric Proton Sink

**Figure 3:**
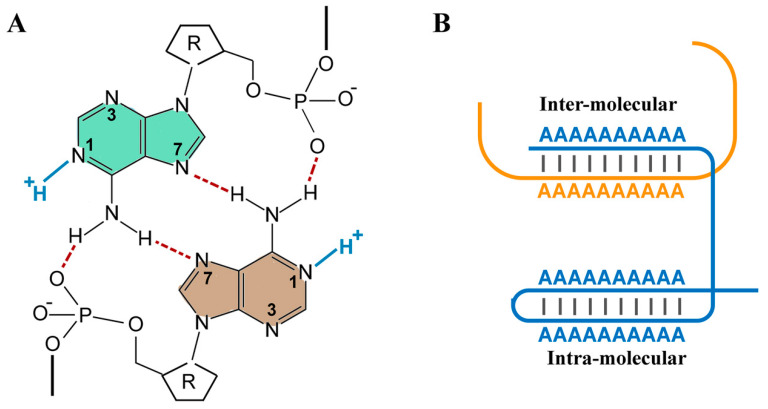
Double helix formation by adenine homo-polymer. (**A**) In double-stranded poly(A), each adenine forms three hydrogen bonds, two H-bonds in the form of N6H… N7, and a H-bond between the N6 hydrogen atom and the phosphate oxygen atom from the opposite chain. Adapted from Zarudnaya et al. [33]. (**B**) Extensive inter- and intra-molecular double-helix formation in poly(A)-rich regions induces a “frozen” state of the RNA.

While RNA is generally studied in the context of information coding, other biophysical properties of the polymer are rarely considered to be of biological significance. Accumulating evidence, however, is gradually reshaping the dominant information-centric view of RNA. In the nucleus, hydrated RNA appears to form a gel-like mesh that in turn regulates the structure and function of chromatin [23]. Another biophysical property of RNA that has been overlooked is the structural capacity of this molecule to function as a polymeric proton sink [24,25]. Both nitrogenous nucleobases and sugar–phosphate backbone could act as a proton sink. While in a neutral pH, adenine and cytosine are unprotonated in single stranded unfolded RNA, and acidification triggers protonation of the imino nitrogens of A and C (pKa ≈ 3.6–4.3) [26]. Conversely, alkalinisation leads to deprotonation of G and U with a pKa value of ≈9.2–9.6 [26]. Based on these pKa values, all nucleobases are expected to be uncharged at biological pH. Phosphodiesters of the RNA backbone, on the other hand, exhibit a pKa value of ≈1 and are mono-anionic at biological pH. In contrast, a terminal phosphate monoester has a higher pKa of ≈6–7 and could oscillate between an unprotonated di-anionic and a protonated mono-anionic form in a physiological pH range. Finally, the pKa of the 2′-hydroxyl group in the ribose moiety varies according to the nucleobase identity and has been estimated to be ≈12.5–15 [27]. While it appears that phosphodiesters are only moieties that could function as a Brønsted base, pKa values in RNA could significantly deviate from these reported values upon assumption of a secondary structure. A typical example of a pKa shift is observed in a lead-dependent RNA enzyme, wherein pKa of A^+^•C mismatch exhibits a shift towards neutrality (pKa ≈ 6.5) [28]. This behaviour is typical of a Class I site wherein the sequestered proton is involved in hydrogen bonding and thus can only be released upon degradation of the RNA [29]. In contrast to a Class I site, sequestered protons are transferrable in a Class II site and contribute to the catalytic activity of ribozyme [30]. Focusing on Class I protonation sites, while nucleobase pKa’s in unstructured regions of RNA have stable pKa’s, Watson–Crick base pairs shift pKa’s away from neutrality, and wobbly A^+^•C pairs shift pKa’s toward neutrality [28]. This protonation tendency of adenine is not confined to the A^+^•C pair. The homo-polymeric form of adenine also functions as an effective proton sink [31,32]. This behaviour becomes apparent between pH 6 and 4, and it is estimated that approximately one equivalent of hydrogen ions is bound per adenine upon titration within this pH range [31]. The single-stranded poly(A) is a right-handed helix mainly stabilised by the stacked array of A bases and by some contribution from cooperative vertical hydrogen bonds between 6-amino groups of the base (… N6H… N6H… N6H) [33]. Upon acidification (pH < 6), protonation of adenines at position N1 triggers transformation of the single-stranded polymer to a double-stranded form [34] (Figure 3). In fact, protonation of adenines and formation of the double-stranded (inter- and intra-polymeric) poly(A) could be so extensive as to induce aggregation of poly(A) tracts in a “frozen” state [35]. Therefore, poly(A) homo-polymers could operate as an efficient proton sink between pH 4–6 [32]. While accurate measurement of pH within IMS is a challenging task and subject to inaccuracies [36], a recorded ΔpH of 1.0–1.2 pH units [37,38] against a reported pH value of 7.4 in the vicinity of ATP synthase in the matrix side [39] suggest that [H^+^] within IMS could approach a minimum concentration of 10^−6^ M. This is, however, a conservative calculation because the concentration of free protons in the solvent is at least an order of magnitude lower than the membrane-associated [H^+^] [4,5]. Therefore, the total concentration of H^+^ (≥10^−5^ M) is expected to be within the buffering capacity of poly(A) homo-polymers. From an evolutionary perspective, it is curious that the nucleobase which functions as an energetic currency (ADP) is also a potent proton acceptor in a polymeric form thereby facilitating deployment of an effective RNA-based proton sink to amplify mitochondrial capacity for ATP synthesis. Finally, the quantitative basis of the proposed revised chemiosmosis theory will be addressed in the next section by focusing on the enzymatic activities of PNPase and the input parameters, ADP and Pi.

## 4. Quantitative Underpinnings of Revised Chemiosmotic Theory

Both in generalised and localised versions of chemiosmosis, protons exported into IMS are assumed to be immediately accessed by F_0_F_1_-ATP synthase. ADP and Pi are supplied to F_0_F_1_-ATP synthase in a manner proposed to be somewhat uncoupled from Δ*p.* Hence, there are no assumed mechanisms to assure that an optimum concentration of Pi and ADP is reached upon activation of the electron transport chain and establishment of Δ*p*. In a revised version of chemiosmosis as proposed herein, an RNA-based proton sink composed of poly(A) homopolymer synthesised by PNPase traps the exported protons in IMS according to Equation (1):

(1) $$n{ADP}^{3-}+nH^{+}\overset{PNPase}{\to }\;{\left({AMP}^{2-}\right)}_{n}.{nH}^{+}+n{{HPO}_{4}}^{2-}\;$$

As an outcome of this stage, ADP and H^+^ are sequestered in poly(A) in an equimolar ratio, and Pi is generated in a 1:1 ratio with H^+^. Once the concentration of inorganic phosphate exceeds a threshold level, PNPase exhibits exonuclease activity according to Equation (2):

(2) $${\left({AMP}^{2-}\right)}_{n}.{nH}^{+}+n{{HPO}_{4}}^{2-}\overset{PNPase}{\to }\;n{ADP}^{3-}+nH^{+}$$

The outcome of this stage is the consumption of Pi and the release of ADP/H^+^ in an equimolar ratio as per Equation (2) (Figure 3). ADP/H^+^ and the remaining Pi will then be utilised by ATP synthase to generate ATP according to Equation (3):

(3) $$n{ADP}^{3-}+nH^{+}+n{{HPO}_{4}}^{2-}\overset{ATP\;synthase}{\to }{nATP}^{4-}\;$$

The model predicts a delay between the activity of ETC and synthesis of ATP as a mechanism to synchronise the availability of ADP and Pi to Δ*p*. Such delay (Δt) is a function of the time that it takes for accumulation of inorganic phosphate in IMS to reach the threshold level for switching on the exonuclease activity of PNPase:

(4) $$If\;\int_{0}^{t}\frac{d{\left[Pi\right]}^{IMS}}{dt}dt>{\left[Pi\right]}_{PNPase}^{threshold}\;\overset{then}{\Rightarrow }\;\left\{\begin{array}{c} \Delta t=t\\ PNPase:exonuclease \end{array}\right.\;$$

Inorganic Pi released from nucleotide diphosphates upon polymerisation by PNPase (see Equation (1)) combined with that imported from the cytoplasmic pool [40] (Pi^cyto^) and Pi exported from the mitochondrial matrix [41] (Pi^matrix^) will combine to raise the concentration of Pi above a threshold level ([Pi]^threshold^).

(5) $${\left[Pi\right]}^{threshold}={Pi}^{PNPase}+{Pi}^{cyto.}+{Pi}^{matrix}\;$$

Consumption of Pi during phosphorolysis by ATP synthase will then complete the cycle as the exonuclease activity of PNPase will switch to a polymerising activity once the concentration of Pi falls below a threshold level after a period of Δt′:

(6) $$If\;\int_{t}^{t}\frac{d{\left[Pi\right]}^{IMS}}{dt}dt<{\left[Pi\right]}_{PNPase}^{threshold}\;\overset{then}{\Rightarrow }\;\left\{\begin{array}{c} {\Delta t}^{\prime }=t^{\prime }-t\\ PNPase:polymerase \end{array}\right.\;$$

Here, Δt′ refers to the time available for the release of H^+^ via degradation of RNA and a concomitant synthesis of ATP by ATP synthase. The proposed revised model of chemiosmosis extends the original version by introducing an additional IMS-localised RNA-based proton sink as follows:

1. The total proton motive force (∑Δp) is defined as protons entrapped in an RNA-base proton sink (Δ*p*^RNA^: latent Δ*p*) together with free protons (Δ*p*^free^*)* in the IMS released from the proton sink:

(7) $$\sum \Delta p={\Delta p}^{RNA}+{\Delta p}^{free}$$

While Δ*p*^RNA^ is maintained by the polymerising activity of PNPase, Δ*p*^free^ is a temporary proton flux generated by exonuclease activity of PNPase.

2. Total proton motive force (∑Δp) is suggested to be predominantly related to Δ*p*^RNA^ and regulated by the capacity of the proton sink combined with activity of the electron transport chain (ETC):

(8a) $$\left\{\begin{array}{c} \;{∆p}^{RNA}\gg \;{∆p}^{free}\\ \;{∆p}^{RNA}\propto \left[H^{+}\;sink\right]\;\cdot [ETC] \end{array}\right.$$

Validity of the assumption of Δp^RNA^>>Δp^free^ can be shown by calculations which concluded that free protons in a mitochondrial IMS are likely to be fewer than 10 [42]. The second Equation of (8a) is a reference to the activity of the ETC that generates protons to be subsequently sequestered by a H^+^ sink. The capacity of poly(A)-based proton sink, in turn, is regulated by polymerising activity of PNPase:

(8b) $$\left[H^{+}\;sink\right]\propto \;{PNPase}^{pol}$$

According to (8b), expansion of the poly(A) pool will enhance the capacity of the proton sink.

Δ*p*^free^, on the other hand, is regulated by exonuclease activity of PNPase which liberates trapped protons:

(8c) $$\sum {\Delta p}^{free}\propto \;{PNPase}^{exo}$$

3. At a higher level, the polymerising activity of PNPase and hence the capacity of the proton sink is programmed by two main energetic inputs, the level of ADP (the substrate for PNPase) and the concentration of Pi (a regulator of exonuclease activity of PNPase). As such, the capacity of the proton sink will grow according to the following Equation:

(9) $$\left[H^{+}\;sink\right]\propto [ADP]/[Pi]$$

Enhanced capacity of the proton sink under conditions of elevated [ADP] and reduced [Pi], such as occurs during intense periods of ATP hydrolysis/phosphorylation characterised by generation of ADP and consumption of Pi, is expected to increase Δ*p*^RNA^ to enhance the efficiency of ATP synthesis by PNPase-mediated conversion of Δ*p*^RNA^ *to* Δ*p*^free^. The proposed role of Pi as the ultimate regulator of ATP synthesis via controlling [H^+^ sink] is bolstered by findings of Bose et al., who dissected the role of Pi in orchestrating oxidative phosphorylation [43]. It was shown that Pi at a concentration of 3 mM reduces ΔpH while amplifying Δψ (i.e., inducing a hyperpolarized state), while ΔG(H^+^) remains essentially constant or increases [43]. An interpretation of this finding is expansion of Δ*p*^RNA^ in a Pi-dependent manner (see Equation (9)) leading to amplification of Δψ, while an unaltered Δ*p*^free^ would manifest as a constant ΔG(H^+^).

Finally, it is curious to explore whether energetic calculations based on the proposed revised chemiosmosis agree with that of the original version of the theory. According to the original theory, the synthesis of ATP requires a proton motive force of 210 mV:

∆p=∆E−60∆pH≈210 mV

To establish a correlation between Δ*p* of the original chemiosmosis theory and Δ*p*^RNA^ of the revised version, it is necessary to estimate the maximum capacity for sequestering protons by an RNA-based sink based on a set of specific assumptions. It can be shown that the estimated maximum capacity of the proton sink requires that [Pi]^threshold^ is entirely generated in a localised manner by polymerising activity of PNPase (see Equations (1) and (5)). A corollary of the localised generation of Pi by conversion of ADP to AMP is that for [*n*] mole of Pi generated via this mechanism, [*n*] mole of H^+^ will be sequestered based on Equation (10):

(10) $$\left\{\begin{array}{c} {\left[Pi\right]}^{PNPase}\to {\left[Pi\right]}^{Threshold}\\ {{[H}^{+}]}^{trapped}{=[Pi]}^{PNPase}={\left[Pi\right]}^{threshold} \end{array}\right.$$

In this scenario, the concentration of [H^+^]^trapped^ and [Pi]^PNPase^ will increase in an equimolar ratio, and the maximum concentration of [H^+^]^trapped^ will be reached as [Pi] approaches a threshold value. In all other scenarios (e.g., importing Pi from the cytoplasm or from the mitochondrial matrix), [Pi]^threshold^ will be reached prior to expansion of the proton sink, triggering exonuclease activity of PNPase and releasing the [H+]^trapped^.

(11) $$\left\{\begin{array}{c} {\left[Pi\right]}^{PNPase}+{Pi}^{cyto.}+{Pi}^{matrix}\;\to {\left[Pi\right]}^{Threshold}\\ {{[H}^{+}]}^{trapped}{=[Pi]}^{PNPase}={\left[Pi\right]}^{threshold}{-(Pi}^{cyto.}+{Pi}^{matrix}) \end{array}\right.$$

In the circumstance of a localised production of [Pi] via PNPase (as per Equation (10)), the threshold concentration of Pi that triggers exonuclease activity of PNPase is ≈0.1 mM [21]. Therefore, the maximum concentration of trapped H^+^ based on Equation (10) is estimated to be ≈0.1 mM. In this scenario, the release of the trapped H+ (≈0.1 mM) against a reported pH value of 7.4 in the vicinity of ATP synthase in the matrix side [39] and given a minimum proton leakage of 20% (i.e., a conductance efficiency of 0.8) would result in conversion of Δ*p*^RNA^ to a Δ*p*^free^:

$${∆p}^{free}=∆E-60∆pH=∆E-60(7.4-(-\log\left(10^{-4}\times 0.8\right))\approx ∆E+200\;mV$$

To calculate ΔE in the presence of an RNA sink, which is considered a non-dialysing electrolyte, Donnan effect may be utilised. This is particularly relevant, as synthesis of a poly(A) sink occurs with a delay, and it takes minutes to lengthen the poly(A) homopolymers to kb range, whereas mitochondrial ion channels operate at a much higher rate. Under this condition, a reported energetic contribution of 10 mV by membrane potential of state 4 mitochondria via Donnan effect [44] could provide the 210 mV of proton motive force required for ATP synthesis. Based on these calculations, it appears that energetic estimates of the revised chemiosmosis correspond to those of the original version of the theory. Finally, it is necessary to explore how the proposed revised RNA-based chemiosmotic theory interfaces with the known mechanisms of respiratory control.

## 5. Mechanisms of Respiratory Control and the Revised Chemiosmotic Theory

While readers are referred to scholarly reviews detailing respiratory control mechanisms [45,46,47], the overview provided here is a prelude to the discussion that follows. The first mechanism of respiratory control emerged from the observation that addition of ADP stimulates oxygen uptake of isolated mitochondria (i.e., state 3 respiration), leading to a reduction in Δp [48,49]. This phase is followed by inhibition of respiration due to transformation of ADP into ATP (i.e., state 4 respiration) and a concomitant increase in Δp [48,49]. According to classic chemiosmotic theory, this happens because a high Δ*p* inhibits the activity of proton pumps. However, several conflicting lines of evidence [47], including an unchanged mitochondrial membrane potential according to the rate of cell respiration [50], hinted at an alternative molecular basis for the first regulatory mechanism of respiratory control. In the revised chemiosmotic theory, consumption of Pi to generate ATP will induce a switch from exonuclease activity of PNPase to a polymerising activity once the concentration of Pi falls below a threshold level (see Equations (1) and (6)). Therefore, the available ADP will be diverted to the synthesis of poly-(A) homopolymers in IMS rather than generation of ATP in matrix.

The second mechanism of respiratory control emerged from studies by Kadenbach et al., who discovered allosteric inhibition of cytochrome c oxidase at a high ATP/ADP ratio [45,46]. ATP exerts this effect by binding to the matrix domain of subunit IV of cytochrome c oxidase [51]. The allosteric inhibition of cytochrome c oxidase by ATP is switched on by cAMP-dependent phosphorylation of the complex and switched off by Ca^2+^-activated dephosphorylation of the protein [52]. In a dephosphorylated excited state, the activity of the complex increases by five- to ten-fold [53], and generation of reactive oxygen species (ROS) by the electron transport chain occurs at a higher rate [45]. The revised chemiosmotic theory interfaces with and complements the second mechanism of respiratory control at multiple levels. At an effector level, amplified ROS generation is expected to trigger oxidation of RNA. The higher propensity of guanine for oxidisation compared to adenine (≈3 times higher propensity [54]), combined with a higher affinity of PNPase to oxidised RNA [55], means that ROS could potentially accelerate the exonuclease activity of the enzyme, thus invoking a high rate of ATP synthesis. This enhanced activity of cytochrome c oxidase comes at a cost of a somewhat reduced efficiency, as a fraction of ADPs will be oxidised and excluded from the pool of usable dinucleotides. The enhanced activity of cytochrome c oxidase upon availability of calcium is important to complement other mitochondrial impacts of this ion. Calcium facilitates dephosphorylation of phosphorylated pyruvate dehydrogenase, thereby activating the enzyme [56]. Calcium also stimulates the entire oxidative phosphorylation cascade [57] within a specific range of ionic concentration [58]. Therefore, it can be argued that the oxidation of RNA within IMS via ROS, which occurs within seconds, primes mitochondria for amplified activity of the electron transport chain in anticipation of an energised citric acid cycle. On the other hand, the reduced activity of PNPase in a ROS^low^ milieu (i.e., phosphorylated cytochrome c oxidase and a high ATP/ADP ratio) will reduce the measurable component of Δ*p^RNA^* (i.e., Δ*p^free^* as per Equation (8a)), manifesting as a reduced membrane potential in this relaxed state. Based on this discussion, it can be said that the revised chemiosmotic theory complements the first and the second respiratory control mechanisms while providing explanations for the role of unexplained features such as amplified ROS production.

## 6. Challenges and Future Directions

An aspect of the proposal that requires further experimentation is potential association of RNA with the lipid bilayer of mitochondrial inner membrane. While there is no direct evidence for such an association, certain inferences can be made based on published reports. Within mitochondrial cristae, cardiolipin stabilises respiratory chain supercomplexes [59] and the lipid bilayer [60]. Recent studies revealed that cardiolipin and phosphatidylethanolamine segregate to the negatively curved monolayer leaflet facing the crista lumen, while the opposing, positively curved, matrix-facing monolayer leaflet contains, predominantly, phosphatidylcholine [61]. It has been suggested that cardiolipin headgroups may function as a proton trap, increasing the concentration of protons in proximity of ATP synthase [62]. However, anionic lipid headgroups of cardiolipin could also engage in anti-electrostatic hydrogen bonding [63] with other anionic groups (e.g., phosphate) [64,65]. Accordingly, potential association of RNA with cardiolipin by forming anti-electrostatic hydrogen bonds provides an alternative mechanism by which cardiolipin could enrich the proton in vicinity of ATP synthase. Considering the evidence for functional association of PNPase and ATP synthase [15,66], an alternative hypothesis is that PNPase associates with ATP synthase directly or indirectly and thus localises the RNA into vicinity of ATP synthase.

Experimental validation of the revised chemiosmotic hypothesis requires experiments in which critical parameters of the model are rigorously controlled. Aside from elimination of any RNase activity, the critical parameters of the proposed model include the concentrations of Pi and ADP within IMS. Also of importance, the incubation time is determined by the concentration of Pi in IMS (see Equations (4) and (6)). The concentration of free ADP within IMS is negligible compared to that supplied exogenously. Hence, controlling the concentration of Pi appears to be pivotal for experimental validation of the proposed model.

## 7. Concluding Remark

In summary, a revised framework for chemiosmotic production of ATP is proposed herein, which entails an intermediary step of storing the protons generated via ETC in an RNA-based proton sink residing in IMS. The proposed proton sink not only sequesters protons transiently but also coordinates the release of trapped protons with the availability of ADP and inorganic Pi, the key substrates of ATP synthase. Regulation of the proton sink is achieved via bimodal activity of PNPase, which can operate both as a polymerase and an exonuclease by receiving input from inorganic phosphate.

## Data Availability

No new data were created or analyzed in this study.
